# Longitudinal Changes of Cornea Volume Measured by Means of Anterior Segment-Optical Coherence Tomography in Patients with Stable and Progressive Keratoconus

**DOI:** 10.3390/life14020176

**Published:** 2024-01-25

**Authors:** Sabrina Vaccaro, Chiara Vivarelli, Angeli Christy Yu, Nicolò Pecora, Giovanna Lionetti, Raffaella Gioia, Vincenzo Scorcia, Giuseppe Giannaccare

**Affiliations:** 1 Department of Ophthalmology, University Magna Græcia of Catanzaro, 88100 Catanzaro, Italy; sabrina_vaccaro@libero.it (S.V.); nicolo.pecora@studenti.unicz.it (N.P.); lionettigiovanna@libero.it (G.L.); raffaella.gioia@studenti.unicz.it (R.G.); vscorcia@unicz.it (V.S.); 2 Department of Translational Medicine, University of Ferrara, 44121 Ferrara, Italy; chiara.vivarelli@edu.unife.it (C.V.); angelichristy.yu@unife.it (A.C.Y.); 3 Department of Ophthalmology, Ospedali Privati Forlì “Villa Igea”, 47122 Forlì, Italy; 4 Eye Clinic, Department of Surgical Sciences, University of Cagliari, Via Università 40, 09124 Cagliari, Italy

**Keywords:** keratoconus, cornea, AS-OCT, corneal volume, corneal thickness

## Abstract

Keratoconus is a corneal disease which results in progressive thinning and protrusion of the cornea leading to irregular astigmatism. The purpose of this study was to evaluate longitudinal changes in corneal volume (CV) occurring over time in keratoconus eyes. Consecutive patients affected by keratoconus were evaluated by means of anterior segment-optical coherence tomography (AS-OCT) at two different time points: baseline (T0) and after 1 year (T1). Anterior and posterior refractive value; corneal thickness at the thinnest point (TP) and corneal volume (CV) calculated within discs of 3, 5 and 8 mm of diameter; anterior chamber depth (ACD); and anterior chamber volume (ACV) were obtained. Enrolled patients were divided into 3 groups (groups 1, 2, 3) according to the increasing disease severity and into 2 groups (groups A, B) according to the progression or stability of the disease. Overall, 116 eyes of 116 patients (76 males and 40 females, mean age 34.76 ± 13.99 years) were included. For the entire group of keratoconus patients, in comparison with T0, mean TP decreased at T1 from 458.7 ± 52.2 µm to 454.6 ± 51.6 µm (*p* = 0.0004); in parallel, mean value of CV calculated at 5 mm and 8 mm decreased significantly (from 10.78 ± 0.8 at T0 to 10.75 ± 0.79 at T1 (*p* = 0.02), and from 32.03 ± 2.01 mm^3^ at T0 to 31.95 ± 1.98 at T1 (*p* = 0.02), respectively). Conversely, there were no statistically significant differences in CV at 3 mm from T0 to T1 (*p* = 0.08), as well as for ACD and ACV. Regarding the course of the disease, patients belonging to group A showed statistically significant differences from T0 to T1 for TP, and for CV at 3 mm, 5 mm and 8 mm (*p* < 0.0001, *p* < 0.0001, *p* < 0.001 and *p* = 0.0058 respectively). There were no statistically significant differences for ACD (*p* = 0.6916) and ACV calculated at 3, 5 and 8 mm (*p* = 0.7709, *p* = 0.3765, *p* = 0.2475, respectively) in group A. At the same time, no statistically significant differences for ACD (*p* = 0.2897) and ACV calculated at 3, 5 and 8 mm (*p* = 0.9849, *p* = 0.6420, *p* = 0.8338, respectively) were found in group B. There were statistically significant positive correlations between changes of TP and CV at 3 mm (r = 0.6324, *p* < 0.0001), 5 mm (r = 0.7622, *p* < 0.0001) and 8 mm (r = 0.5987 *p* < 0.0001). In conclusion, given the strong correlation with TP, CV might be considered an additional AS-OCT parameter to be used in association with conventional parameters when detecting longitudinal changes in keratoconic eyes.

## 1. Introduction

Keratoconus is a complex corneal disease, associated with both genetic and environmental factors [1,2]. Once considered non-inflammatory, its etiopathogenesis has now been related to pro-inflammatory pathways [3,4,5]. It is characterized by central corneal thinning and protrusion that lead to progressive irregular astigmatism [1,2]. The majority cases, accounting for 90% of the total, are binocular, often characterized by asymmetrical progression in both eyes [6,7]. The alteration in the curvature of the cornea results in the generation of substantial levels of higher order aberrations, leading to a significant reduction in visual quality [8]. In 2015, the Global Consensus on Keratoconus and Ectatic Diseases highlighted the role of changes occurring in the posterior corneal surface and in corneal thickness for the diagnosis of keratoconus [9]. Alterations of both disposition and composition of collagen lamellae have been shown in eyes with keratoconus. This alteration leads to loss of corneal rigidity and, consequently, corneal protrusion and thinning [10,11]. It has been proposed that several factors, including localized changes in proteolitic enzyme activity and pro-inflammatory citokines and a reduced production of collagen I, are associated with breaks in Bowman’s layer and may simultaneously also promote the unraveling of collagen fibrils, lamellar slippage and the disinsertion of lamellae from their anchoring sites in Bowman’s layer [12,13,14,15,16]. When comparing keratoconus with normal corneas, the collagen fibrillar mass is unevenly distributed, particularly at the apex of the cone, and the organization of the stromal lamellae is drastically altered [12].

The diagnosis of keratoconus is reached thanks to corneal imaging that is able to provide different levels of details, from corneal topography and pachymetry to anterior segment-optical coherence tomography (AS-OCT). These instruments are essential when assessing the course of the pathology. Corneal topography facilitates a comprehensive evaluation of the precise shape, size and location of keratoconus. The analysis of corneal ectasia involves the assessment of corneal thickness through the use of corneal pachymetry [9,17]. Furthermore, the identification of keratoconus has undergone progressive improvements in recent years, passing from basic assessments of the anterior corneal surface to precise three-dimensional structural analysis of the entire cornea [18]. The concept of considering the cornea as a solid entity possessing a distinct volume has been put forth as a prospective and valuable clinical parameter for the identification and monitoring of frank keratoconus, as well as for patients with subclinical manifestations [19,20]. The clinical application of AS-OCT, a non-contact technique that follows the principles of low-coherence interferometry, has enabled the determination of structural corneal changes that occur in keratoconus as the disease progresses [21]. Through the examination of the alteration that occurs in the corneal microarchitecture of eyes affected by keratoconus, the disease’s severity can be determined [22].

Though corneal volume (CV) measured by means of AS-OCT is sometimes used in clinical and research practice to provide additional information about changes involving posterior cornea, the real clinical impact of this parameter is not known [23,24]. Several studies have reported a reduction in the corneal thinnest point (TP) in patients with keratoconus compared with normal eyes, which is associated with the increase of corneal curvature and the progression of the disease [19,25,26,27]. However, the reduction of CV was shown only in advanced stages. In particular, Morishige et al. have demonstrated that loss of CV occurs after the reduction of TP, suggesting that it is affected by the stromal degradation that occurs only in the advanced stage of keratoconus [28].

Since longitudinal changes of CV in keratoconus eyes have yet to be investigated, we sought to evaluate, over time, these modifications in patients with both stable and progressive keratoconus.

## 2. Materials and Methods

**Figure 1:**
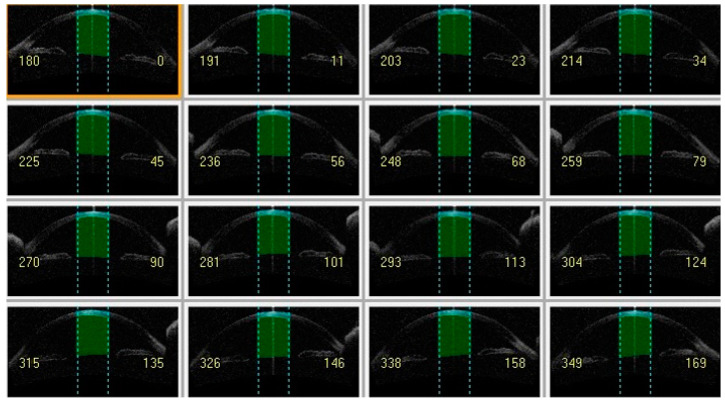
Anterior segment-optical coherence tomography showing cornea volume at 3 mm (light green) and anterior chamber volume at 3 mm (dark green).

**Figure 2:**
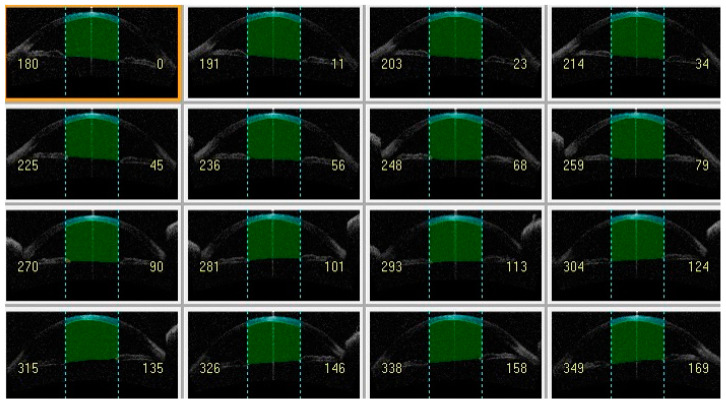
Anterior segment-optical coherence tomography showing cornea volume at 5 mm (light green) and anterior chamber volume at 5 mm (dark green).

**Figure 3:**
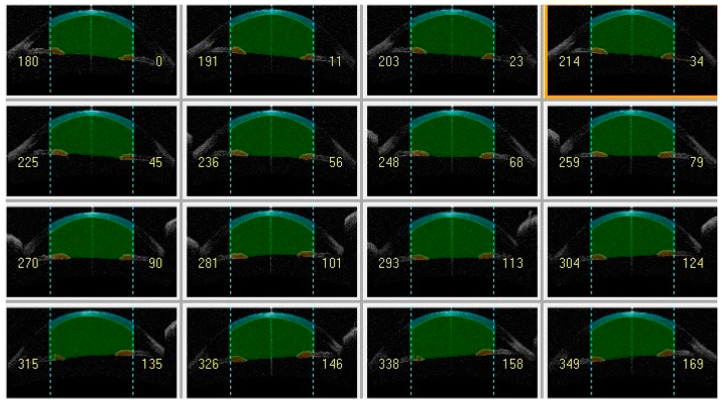
Anterior segment-optical coherence tomography showing cornea volume at 8 mm (light green) and anterior chamber volume at 8 mm (dark green).

This institutional retrospective cohort study reviewed the medical records of all keratoconus eyes followed at a tertiary referral center (Department of Ophthalmology, University of Magna Graecia, Catanzaro, Italy) from April 2012 to December 2021. The study followed the principles of the 1964 Declaration of Helsinki and was approved by the local ethics committee (Comitato Etico Regione Calabria—Sezione Area Centro). A detailed informed consent form for study participation was signed by all patients during a routine control visit. Inclusion criteria were a diagnosis of keratoconus and the presence of two examinations with good quality corneal imaging one year apart. Data from each subject were examined at least twice by different ophthalmologists to confirm the diagnosis. The presence of keratoconus was determined by a slit lamp examination, which included signs such as stromal thinning, Vogt’s streaks, Fleischer’s rings, and tomographic findings suggestive of keratoconus. The exclusion criteria for this study included any history of ocular surgery, particularly eyes that had undergone keratoplasty; systemic or ocular disease (excluding keratoconus); or use of ocular medications (excluding antihistamines). Patients who were wearing contact lenses were instructed to discontinue their use 48 h prior to their scheduled visit. Tomographic maps for keratometry and pachymetry values and AS-OCT scans for CV at different diameters, namely 3 mm (Figure 1), 5 mm (Figure 2) and 8 mm (Figure 3) centered on the corneal apex, were obtained using Casia 1 (Tomey Corp., Nagoya, Japan) at baseline (T0), and after 1 year (T1) and 2 years (T2).

Thanks to the three-dimensional image of the anterior segment, the calculations of anterior chamber depth (ACD) and anterior chamber volume (ACV) were also performed. Data were collected by the same examiner (NP) and analyzed independently by an additional operator (SV).

Each eye with keratoconus was staged in terms of severity of the disease according to the steepest keratometry (K) values, using a simple keratoconus classification, and according to the progression of the disease evaluated as a reduction of TP in 1 year. With regard to the former classification, eyes with a steepest K value lower than 45 D were classified as mild (group 1), those with a K value between 45 and 52 D as moderate (group 2) and those with K value higher than 52 D as severe (group 3) [25]. With regard to the latter classification, eyes with an increase of K values > 1 D and/or pachymetry with a ≥2% decrease in TP in 1 year were classified as progressive keratoconus (group A) while those with no/lower increase were classified as stable (group B) [29,30]. During the period of observation, keratoplasty was not necessary for any of the eyes that were examined.

### Statistical Methods

Statistical analyses were performed using Prism version 9.4.0 (GraphPad Software Inc., San Diego, CA, USA) and Jamovi version 2.4.1.0 (The jamovi project, Sidney, Australia). Normally distributed data were expressed as mean ± standard deviation (SD), otherwise as median values with interquartile range (IQR). Parametric and nonparametric tests were chosen based on data normality. The Anderson–Darling and Kolmogorov–Smirnov tests were applied to assess whether data were normally distributed. Student’s *t* test, Mann–Whitney U test, Dunnett’s multiple comparison test and Friedman test were used to compare variables, when appropriate. The Pearson correlation test was used to evaluate the correlation of parameters. A *p*-value less than 0.05 was considered statistically significant.

## 3. Results

The study included 116 eyes of 116 patients (76 males and 40 females, mean age 34.76 ± 13.99 years) who received two consecutive annual examinations. At baseline, 72 eyes were classified as affected by mild keratoconus (group 1), 34 as moderate (group 2) and 10 as severe (group 3). There were no statistically significant differences among groups for age, sex or eye distribution. The mean ± SD steep K values for mild, moderate and severe cases were 45.70 ± 1.60 D, 50.78 ± 1.42 D and 56.86 ± 1.86 D, respectively. The mean ± SD values for CV at the different diameter discs (3, 5, and 8 mm) are shown in Table 1.

**Figure 4:**
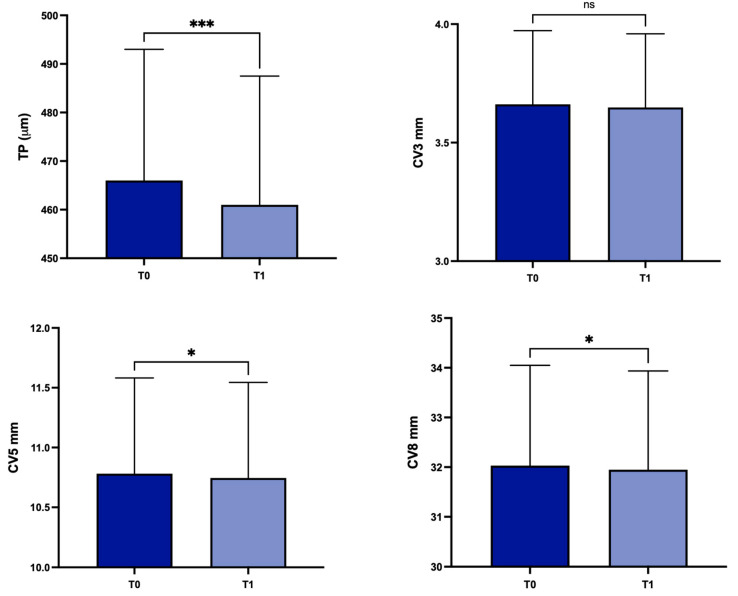
Values of corneal thickness at the thinnest point, and corneal volume at baseline and 1 year later in the overall study population. TP: corneal thickness at the thinnest point; CV3: corneal volume at 3 mm diameter; CV5: corneal volume at 5 mm diameter; CV8: corneal volume at 8 mm diameter; T0: baseline; T1: 1 year later; ns, not significant; * *p* < 0.05; *** *p* < 0.001.

For the entire group of keratoconus patients, in comparison with T0, mean TP decreased at T1 from 458.7 ± 52.2 µm to 454.6 ± 51.6 µm (*p* = 0.0004); in the same period, statistically significant differences were detected regarding CV at 5 mm and 8 mm (from 10.78 ± 0.8 mm^3^ to 10.75 ± 0.79 mm^3^, *p* = 0.02, and from 32.03 ± 2.01 mm^3^ to 31.95 ± 1.98 mm^3^, *p* = 0.02, respectively). Conversely, there were no statistically significant differences in CV at 3 mm from T0 (3.66 ± 0.31 mm^3^) to T1 (3.64 ± 0.31 mm^3^) (*p* = 0.08) (Figure 4), or for ACD (*p* = 0.2568) and ACV calculated at 3, 5 and 8 mm (*p* = 0.8464, *p* = 0.4627, *p* = 0.3714 respectively).

There were positive correlations that were statistically significant between the average TP and the CV at 3 mm (r = 0.918; *p* < 0.001), 5 mm (r = 0.891; *p* < 0.001) and 8 mm (r = 0.788; *p* < 0.001) (Table 2).

**Figure 5:**
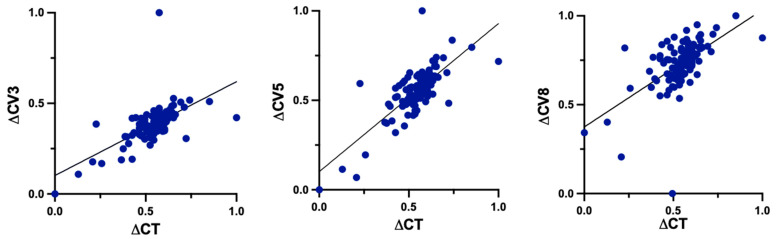
Correlations between changes (Δ) of TP and CV at 3 mm, 5 mm and 8 mm. TP: corneal thickness at the thinnest point; CV3: corneal volume at 3 mm diameter; CV5: corneal volume at 5 mm diameter; CV8: corneal volume at 8 mm diameter.

There were statistically significant positive correlations between changes of TP and CV at 3 mm (r = 0.6324, *p* < 0.0001), 5 mm (r = 0.7622, *p* < 0.0001) and 8 mm (r = 0.5987, *p* < 0.0001) (Figure 5).

**Figure 6:**
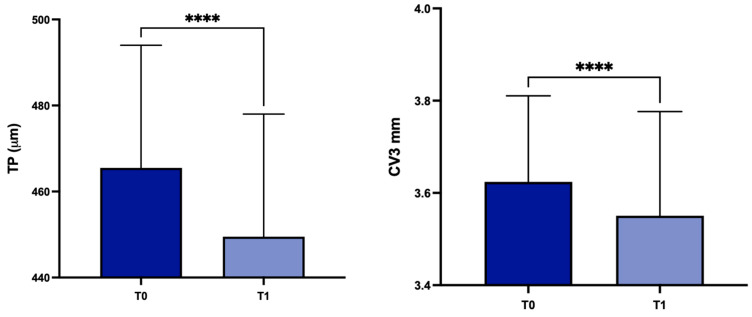

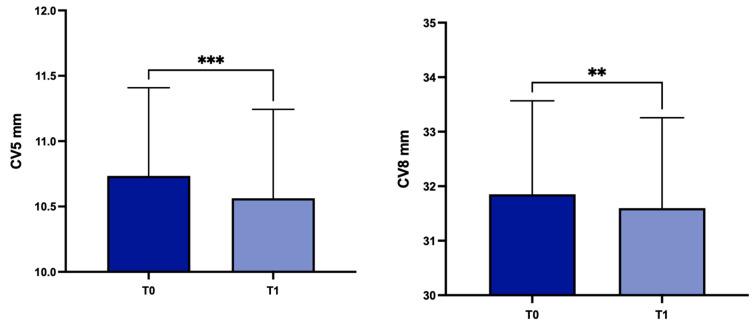
Values of TP, CV3, CV5 and CV8 values at T0 and in the subgroup A. TP: corneal thickness at the thinnest point; CV3: corneal volume at 3 mm diameter; CV5: corneal volume at 5 mm diameter; CV8: corneal volume at 8 mm diameter; T0: baseline; T1: 1 year later; ** *p* < 0.01, *** *p* < 0.001, **** *p* < 0.0001.

Concerning patients with stable (group B; *n* = 86) or progressive (group A; *n* = 30) disease, there were statistically significant differences in group A for TP, CV at 3 mm, 5 mm and 8 mm (*p* < 0.0001, *p* < 0.0001, *p* < 0.001 and *p* = 0.0058 respectively) (Figure 6).

In patients belonging to group B, there were no statistically significant differences in TP or in CV at 3 mm, 5 mm and 8 mm (*p* = 0.4601, *p* = 0.3275, *p* = 0.8482 and *p* = 0.5574 respectively). There were no statistically significant differences for ACD (*p* = 0.6916) or for ACV calculated at 3, 5 and 8 mm (*p* = 0.7709, *p* = 0.3765, *p* = 0.2475 respectively) in group A. At the same time, no statistically significant differences for ACD (*p* = 0.2897) or for ACV calculated at 3, 5 and 8 mm (*p* = 0.9849, *p* = 0.6420, *p* = 0.8338 respectively) were found in group B.

## 4. Discussion

The Global Consensus on Keratoconus and Ectatic Diseases highlighted the important role of the assessment of alterations in the posterior corneal surface and corneal thickness when diagnosing keratoconus [8]. The recent adoption of AS-OCT for the assessment of CV might offer additional insights into alterations affecting the cornea [20,23]. AS-OCT has the advantage of acquiring three-dimensional images of the anterior segment of the eyes, in addition to cross-sectional images [21]. Significant changes in TP and CV have been reported between patients with subclinical and clinical keratoconus in comparison with a control group [23]. The combined use of pachymetric and volumetric data has been shown to offer a more effective assessment of corneal structure in patients with keratoconus, exhibiting favorable levels of sensitivity and specificity when identifying both clinical and subclinical cases [24,25,26,27,28,31]. A previous study has documented a considerable reduction in CV in patients with moderate and severe keratoconus, with the most pronounced decrease observed in the central and para-central regions. This implies that the decrease in CV could be a result of stromal degradation, a process that happens in the later stages of the disease [32]. A recent study created a quantitative staging method for keratoconus using AS-OCT and demonstrated a robust concordance with the already established classification systems. This staging method, named STEP is determined by the stromal overall minimum thickness (ST) and epithelium overall standard deviation (EP). Integrating stromal and corneal epithelial data with the use of AS-OCT has the potential to further improve the treatment and management of keratoconus [33]. Cavas-Martínez et al. conducted a study that provided evidence of a notable decrease in CV during the early stages of keratoconus. Furthermore, the study revealed that the extent of this loss becomes more pronounced as the severity of the disease increases, as determined by the Amsler–Krumeich grading system [32,34].

In accordance with these findings, our study demonstrates a statistically significant decrease over time of TP in patients with keratoconus. At the same time, statistically significant differences were detected regarding CV at 5 mm and 8 mm (but not at 3 mm) from T0 to T1. This discrepancy may be attributed to the fact that a larger diameter allows for a higher likelihood of analyzing the corneal area in which the cone is typically located, which is situated in the middle periphery rather than the central region [35]. Similarly, Cui et al. analyzed 48 patients, a control group of 29 individuals with myopic astigmatism and 19 patients who had subclinical keratoconus. The corneal assessment was conducted using the Pentacam Scheimpflug system, in addition to central corneal thickness they considered CV at 3 mm, 5 mm and 7 mm. They found that subclinical keratoconus and normal corneas differed significantly with regard to all parameters, with the exception of CV3 and CV5. The study utilized partial least squares (PLS) analysis to develop models, taking into account thickness and corneal volume values. The resulting model had a sensitivity of 79.3% and a specificity of 94.7%. It appears that the combination of corneal thickness and volume parameters distinguishes keratoconus corneas from normal corneas more effectively than a single parameter [31]. In a previous study Ambrósio et al., in order to investigate corneal architecture, presented novel corneal tomography parameters obtained from the Pentacam Comprehensive Eye Scanner. Specifically, they analyzed the distribution of corneal volume and the spatial profile of corneal thickness, calculated at different diameters of CV from 1.0 to 7.0 mm with 0.5 mm steps, in order to distinguish keratoconic from healthy corneas. Keratoconic eyes are characterized by thinner diameter and volume, with a more abrupt increase in these parameters from the thinnest point to the periphery, compared with normal eyes [24]. A recent study assessed the precision of various corneal parameters in identifying keratoconus by employing a dual Scheimpflug–Placido system. The study found that the corneal volume at 10 mm did not show any significant differences between the keratoconus group and the healthy group [36].

In contrast with prior investigations, our research exclusively focused on patients diagnosed with keratoconus. Within the subgroup of patients with progressive disease (group A), there were statistically significant differences observed in either thickness or volume measurements over time. On the contrary, no differences were detected in patients with stable disease (group B). Furthermore, strong correlations have been demonstrated between TP (both mean values and changes from T0 to T1) and all of the types of CV (3 mm, 5 mm and 8 mm) in the entire population of patients irrespective of the progression of the disease.

Knowing the cornea’s volume could be useful to assess the progression of the KC. However, it is important to note that CV has been measured through the utilization of several clinical examination methods, such as a Scheimpflug camera and anterior segment-optical coherence tomography (AS-OCT). In particular, Ambrósio et al. employed the Pentacam Comprehensive Eye Scanner. Corneal thickness measurements were taken at the point of minimum thickness for each eye. The mean thickness values of the points on 22 hypothetical circles, centered on the thinnest point, were computed. These calculations were used to generate the spatial profile of corneal thickness for the creation of the corneal volume distribution. The percentage increase in volume was determined for each position by applying a mathematical formula to the initial volume of 1 mm [24]. Cervin õ et al. used a software to precisely simulate a surface using the collected data points obtained from corneal topography and ultrasonic topographic pachymetry. The volume underneath both the front and back sides of the cornea was then calculated [20]. Another study utilized raw topographic data from the Sirius system to enable morphogeometric modeling of the cornea with the assistance of CAD tools [32]. All of these studies used different software to obtain CV as, without the utilization of these programs, incorporating the parameter into clinical practice becomes challenging. In our study the calculation of CV was performed using the SS-OCT Viewer program developed by Tomey. The area corresponding to the cornea was automatically delineated in the 16 AS-OCT pictures, and the volume of interest was then determined. This method had been used by Morishige et al., who found that stromal degradation, which only happens in the advanced stage of keratoconus, has an impact on the loss of CV [28]. The primary limitation of this method is the imprecise automated segmentation. The AS-OCT software (Tomey Link Exam Viewer Version 6P.1) independently generates the demarcated area. Therefore, it is of greatest importance to have control over segmentation. Simultaneously, it is crucial to verify that the examination is centered on the corneal apex in order to establish a standardized assessment.

Furthermore, OCT provides evaluation of the changes in advanced cases of keratoconus, when the accuracy of corneal topography for reliability is reduced. In particular, when severe stromal lesions are present and a patient is undergoing keratoplasty for KC, the AS-OCT analysis is critical for determining the optimal strategy, particularly with regard to the surgical approach and methodology utilized during deep anterior lamellar keratoplasty (DALK) [17]. A previous study into the predictors that influence the formation and type of big bubbles (BB) during DALK revealed that the existence of posterior scars signifies a noteworthy risk factor for type 2 BB formation; in these cases, manual techniques should be considered to reduce the risk of penetrating keratoplasty (PK) conversion and prevent intraoperative complications [17].

Although our study is the first to investigate the longitudinal changes of CV in a large keratoconus population, it is important to acknowledge the inherent limitations of our study that deserve mentioning. Firstly, a control group of healthy subjects without corneal abnormalities has not been included in the analysis. Secondly, in order to obtain a sufficiently wide group of patients with progressive keratoconus, a cut-off value that is lower than is typical was used for the reduction of TP in 1 year to identify patient belonging to this group.

In conclusion, AS-OCT is progressively assuming a crucial role in the examination of corneal modifications among patients diagnosed with keratoconus. Given the positive correlations between TP and CV, the latter could be a useful parameter, but further research is required to elucidate the diagnostic performance of CV when evaluating patients affected by keratoconus.

## Figures and Tables

**Table 1:** Baseline corneal volume at three diameters.

	All (*n* = 116)	Group 1 (*n* = 72)	Group 2 (*n* = 34)	Group 3 (*n* = 10)
Corneal volume 3 mm Ø (mm^3^) mean ± SD	3.67 ± 0.29	3.77 ± 0.25	3.53 ± 0.27	3.24 ± 0.28
Corneal volume 5 mm Ø (mm^3^) mean ± SD	10.79 ± 0.75	11.01 ± 0.70	10.55 ± 0.73	9.92 ± 0.90
Corneal volume 8 mm Ø (mm^3^) mean ± SD	32.05 ± 1.90	32.38 ± 1.89	31.70 ± 2.01	30.68 ± 2.31

SD: standard deviation.

**Table 2:** Correlation matrix among corneal thickness at the thinnest point, and corneal volume at different diameters.

Correlation Matrix					
		TP	CV 3 mm	CV 5 mm	CV 8 mm
TP	Pearson’s r	-			
	df	-			
	*p*-value	-			
CV 3 mm	Pearson’s r	0.918	-		
	df	114	-		
	*p*-value	<0.001	-		
CV 5 mm	Pearson’s r	0.891	0.971	-	
	df	114	114	-	
	*p*-value	<0.001	<0.001	-	
CV 8 mm	Pearson’s r	0.788	0.888	0.960	-
	df	114	114	114	-
	*p*-value	<0.001	<0.001	<0.001	-

TP: Corneal thickness at the thinnest point; CV3: Corneal volume at 3 mm diameter; CV5: Corneal volume at 5 mm diameter; CV8: Corneal volume at 8 mm diameter.

## Data Availability

Data are contained within the article.
